# Chromosome-level haplotype-resolved genome assembly of the linguliform brachiopod *Discradisca antillarum* (d'Orbigny, 1845)

**DOI:** 10.1093/g3journal/jkaf233

**Published:** 2025-10-01

**Authors:** Nickellaus G Roberts, Sarah M Tweedt, Christopher Meyer, Torsten H Struck, Kevin M Kocot

**Affiliations:** Department of Biological Sciences, The University of Alabama, Tuscaloosa, AL 35487, United States; Department of Invertebrate Zoology, Smithsonian National Museum of Natural History, Smithsonian Institution, Washington, DC 20560, United States; Department of Invertebrate Zoology, Smithsonian National Museum of Natural History, Smithsonian Institution, Washington, DC 20560, United States; Natural History Museum, University of Oslo, Oslo 0562, Norway; Department of Biological Sciences, The University of Alabama, Tuscaloosa, AL 35487, United States; Alabama Museum of Natural History, The University of Alabama, Tuscaloosa, AL 35487, United States

**Keywords:** genome assembly, Brachiopoda, lingulata, Lophotrochozoa, Spiralia, chromosome

## Abstract

Brachiopods are a group of shelled, filter-feeding marine invertebrates. Though they superficially resemble bivalve mollusks, brachiopods fall within the clade Lophophorata (Brachiopoda, Bryozoa, Phoronida) and possess an abundant and diverse fossil record spanning more than 500 million years. This long evolutionary history makes Brachiopoda particularly important for understanding the evolution of the morphologically disparate superphylum Lophotrochozoa. Brachiopods also stand to provide insight into the evolution of biomineralization, as most lineages produce skeletons of calcium carbonate while linguliforms use calcium phosphate. However, genomic resources for brachiopods are limited. To enable comparative and evolutionary genomic studies of this clade, we generated a chromosome-level assembly for the discinid brachiopod *Discradisca antillarum*. Sequencing was performed using PacBio HiFi and Hi-C to produce both a primary (*N* = 8) and haplotype-resolved (2*N* = 16) assembly. The primary assembly comprises 208 scaffolds with the 8 chromosome-level scaffolds representing 96.6% of the genome. The primary assembly has a BUSCO genome completeness score of 95.5% (94.4% single copy, 1.0% duplicated, 0.9% fragmented). Annotation of protein-coding genes yielded 29,208 genes with a BUSCO protein completeness score of 95.6% (87.6% single copy, 8.0% duplicated, 2.8% fragmented). Comparative synteny between *D. antillarum* and other lophotrochozoans shows that, at the macrosyntenic level, the genome conserves all proposed ancestral lophotrochozoan fusion-with-mixing events while also revealing new fusions involving several bilaterian ancestral linkage groups. The genome of *D. antillarum* will enable significant insights into brachiopod evolution.

## Introduction

Brachiopods, or “lamp shells,” comprise a phylum of over 850 extant species of shelled, filter-feeding marine invertebrates. Globally distributed in marine habitats (Ye et al. 2021), brachiopods play a key role in many benthic communities (Carlson 2016; Ye et al. 2023). Moreover, brachiopods are among the most significant components of the marine fossil record, resulting in an in-depth understanding of their evolutionary history and changes in diversity through time. Although over 3,000 genera are described from the early Cambrian onwards, 95% of these are extinct and the modern brachiopod fauna represents just a fraction of the group's former diversity (Williams et al. 1997; Carlson 2016; Dulai 2017). The phylum's extant diversity is divided into 3 major clades, Craniiformea, Linguliformea, and Rhynchonelliformea, which differ by characteristics of the attachment of the dorsal and ventral valves and the presence or absence of a fleshy pedicle used for attachment to the substrate or burrowing within it.

Brachiopods belong to the larger clade Lophophorata, which comprises Brachiopoda, Bryozoa, and Phoronida (Nesnidal et al. 2013; Temereva 2017), and is part of the morphologically heterogeneous animal “superphylum” Lophotrochozoa (≈Spiralia), which also includes the phyla Mollusca, Annelida, Platyhelminthes, and Rotifera among others. Although lophophorate monophyly has been questioned (Passamaneck and Halanych 2006; Helmkampf et al. 2008; Paps et al. 2009; Kocot et al. 2017; Khalturin et al. 2022), most recent phylogenomic studies support the monophyly of the clade (Nesnidal et al. 2013; Laumer et al. 2019; Marlétaz et al. 2019). However, these studies conflict in their placement of Brachiopoda within Lophophorata and Lophophorata within the superphylum Lophotrochozoa.

Improved genomic resources for Brachiopoda could help resolve brachiopod and lophophorate phylogenetic placement. However, genomic resources are limited for this phylum, with only the genome of *Lingula anatina* from the family Lingulidae available to date, from which an increased understanding of brachiopod biomineralization, evolution, and development has been gained (Luo et al. 2015; Gerdol et al. 2018; Lewin et al. 2025). In order to further study the genomics of this historically rich and widespread group, we sequenced the genome of *Discradisca antillarum* (d'Orbigny, 1845), a species of sessile linguliform brachiopod in the family Discinidae. *D. antillarum* inhabits hard benthic substrates from tropical and subtropical regions ranging from Florida, throughout the Gulf of Mexico to Brazil (Tunnell 1982), and, as a member of Discinidae, has a rich fossil record (Dulai 2017). Because of these interesting characteristics, we sequenced, annotated, and analyzed the chromosome-level genome of this species to facilitate more in-depth study of the evolution and genomic organization of this phylum.

## Methods

### Specimen collection

Specimens of *D. antillarum* were collected from an Autonomous Reef Monitoring Structure (ARMS; FTP_2022_ARMS_01) deployed on sand adjacent to an oyster reef for 105 months (November 2013—August 2022) off Fort Pierce, Florida (27.488815, −80.31498) at a depth of 2 m as part of the Fort Pierce 2022 ARMS project (https://geome-db.org/record/ark:/21547/EHr2). Live animals were photographed, and tissues were preserved by flash-freezing or in RNAlater and stored at −80 °C.

### DNA extraction and sequencing

Flash-frozen, cryo-preserved mixed tissues from 1 animal (field code XFTP_0019, USNM:IZ:1750834) were used for high-molecular-weight DNA extraction using the NEB Monarch HMW DNA Extraction Kit. DNA concentration and molecular weight distribution were assessed with an Agilent Fragment Analyzer using the Genomic DNA 50 kb Kit. DNA was frozen on dry ice and sent to Discovery Life Sciences (Huntsville, AL, USA) for Pacific Biosciences HiFi v2.0 library preparation and sequencing on 1 Revio SMRT cell.

Hi-C library preparation was performed in-house on the remaining flash-frozen tissues of the same individual (XFTP_0019) using the Phase Genomics Hi-C Animal Kit KT2030 v.3 with 0.2 g of tissue (macerated directly from frozen material) following the manufacturer's instructions. The resulting library was sequenced on an Illumina NovaSeq S4 flow cell at Azenta (South Plainfield, NJ, USA) with 2×150 bp reads.

Transcriptome data were generated from another entire individual (XFTP_0040, USNM:IZ:1750835) collected from the same ARMS. RNA was extracted using the Omega Bio-Tek RNA MicroElute Kit. RNA concentration was measured using a Thermo Fisher Qubit 3.0 fluorometer with the RNA High Sensitivity kit, purity was assessed by measuring the 260/280 nm absorbance ratio using a Thermo Fisher Nanodrop Lite, and integrity was evaluated using a 1% SB agarose gel. Complementary DNA (cDNA) was synthesized from 1 ng of total RNA using the Clontech SMART-Seq HT kit. An Illumina sequencing library was then prepared in-house using the Takara SMART-Seq HT Plus kit with 5 ng of cDNA. Sequencing library molecular weight distribution and concentration were assessed using an Agilent Fragment Analyzer with the NGS 1–6000 bp Kit. The resulting library was sent to Azenta for sequencing on an Illumina NovaSeq S4 flowcell with 2×150 bp PE reads.

### Genome assembly and scaffolding

The genome of *D. antillarum* was assembled using HiFi reads combined with Hi-C data for scaffolding. Assembly of the HiFi reads was performed using hifiasm 0.13-r308 (Cheng et al. 2021) with inclusion of the Hi-C data, which were first quality trimmed and filtered using trim_galore (https://github.com/FelixKrueger/TrimGalore; –quality 30 –length 50) generating phased contigs and haplotype-resolved unitig graphs. Fasta files for the primary contigs, haplotype-resolved contigs, and both haplotypes were generated from the. gfa file output.

Hi-C reads were aligned to the genome using BWA v.0.7.17-r1188 (Li and Durbin 2009) and curated with SAMtools (v.1.10) (Li et al. 2009; Danecek et al. 2021) for input to HapHiC (Zeng et al. 2024). HapHiC was chosen as opposed to other Hi-C scaffolding methodologies due to its ability to more accurately resolve haplotypes and provide better orientation and ordering of contigs within scaffolds (e.g. YAHS, ALLHic; Zeng et al. 2024). Hi-C reads were filtered using HapHic's recommended filtering (samtools -F 3340, filter_bam HiC.bam 1 (MAPQ > 1) –nm 3). The Hi-C reads were aligned to both haplotypes—the haplotype-phased assembly and the diploid assembly. HapHiC was used to partition contigs and generate a contact map (haphic pipeline assembly.fasta filteredbamfile.bam –threads 16 –max_inflation 3 –correct_nrounds 2 –RE “GATC,GANTC,CTNAG,TTAA”). Manual scaffolding was conducted using Juicebox 2.15 (Durand et al. 2016) to refine the chromosome count and organization.

Following manual curation to determine chromosome number, the full HapHic pipeline was run on the primary assembly, the diploid assembly, and both haplotypes using the estimated chromosome number (*N* = 8, 2*N* = 16), and results were manually curated in Juicebox. The final scaffolding steps were performed using Juicer post v1.2. Each fasta file was then filtered to remove debris using Seqkit v2.7.0 (Shen et al. 2016).

### Genome assembly assessment

Genome completeness was measured using BUSCO v.5.8 (Manni et al. 2021) using the metazoa_odb10 dataset and QUAST v.5.0 (Gurevich et al. 2013). K-mer completeness was measured using meryl v.1.4.1 and merqury v.1.3 (Rhie et al. 2020). The percentage of shared k-mers between haplotypes was calculated using meryl union and meryl intersect (meryl union output union_kmers hap_1 hap_2, meryl intersect [equal-to 1 hap_1] equal-to 1 hap_2 output intersect_kmers) and summarized with meryl statistics.

### Annotation

Repeats in the final assembly were annotated with RepeatModeler 2.0.1 (Flynn et al. 2020) and softmasked with RepeatMasker 4.1.2. For RepeatModeler, a maximum genome sample size of 1 M and the –LTRStruct option were used. For RepeatMasker, the slow and gccalc options were used. The engine used for both programs was rmblast 2.11.0+. Available brachiopod and phoronid transcriptome data including the newly sequenced *D. antillarum* transcriptome were translated with TransDecoder (see “Protein Evidence” folder on Figshare for details) and these plus predicted proteins from the *L. anatina* (GCA_001039355.2) and *Phoronis australis* (GCA_002633005.1) genomes were used as protein evidence for genome annotation with BRAKER 3 (Stanke et al. 2006; Hoff et al. 2019; Gabriel et al. 2021).

### Synteny analysis

Interspecies synteny was inferred based on the chromosomal position of orthologous genes using the SyntenyFinder pipeline (Lewin et al. 2025). Proteomes and genomic features from 5 species were analyzed: *D. antillarum* (Brachiopoda; this study), *Pecten maximus* (Mollusca; GCF_902652985.1), *Lineus longissimus* (Nemertea; GCF_910592395.1, *Watersipora subatra* (Bryozoa; GCF_963576615.1), and *Branchiostoma floridae* (Chordata; GCA_000003815.2). Single-copy orthologous genes, defined as orthogroups containing exactly 1 sequence from each species, were identified using OrthoFinder v2.5.4 (Emms and Kelly 2019). A dataset of 24 bilaterian ancestral linkage groups (ALGs) was assembled and each orthogroup (i.e. gene) was assigned to an ALG based on the results of Simakov et al. (2022) and Lewin et al. (2025). Any genes that could not be assigned to an ALG were not considered further. SyntenyFinder (Lewin et al. 2025) was used to generate genomic coordinate and karyotype files and produce riparian plots tracing the bilaterian ALGs across phyla.

## Results and discussion

### Genome assembly and annotation

**Fig. 1.**
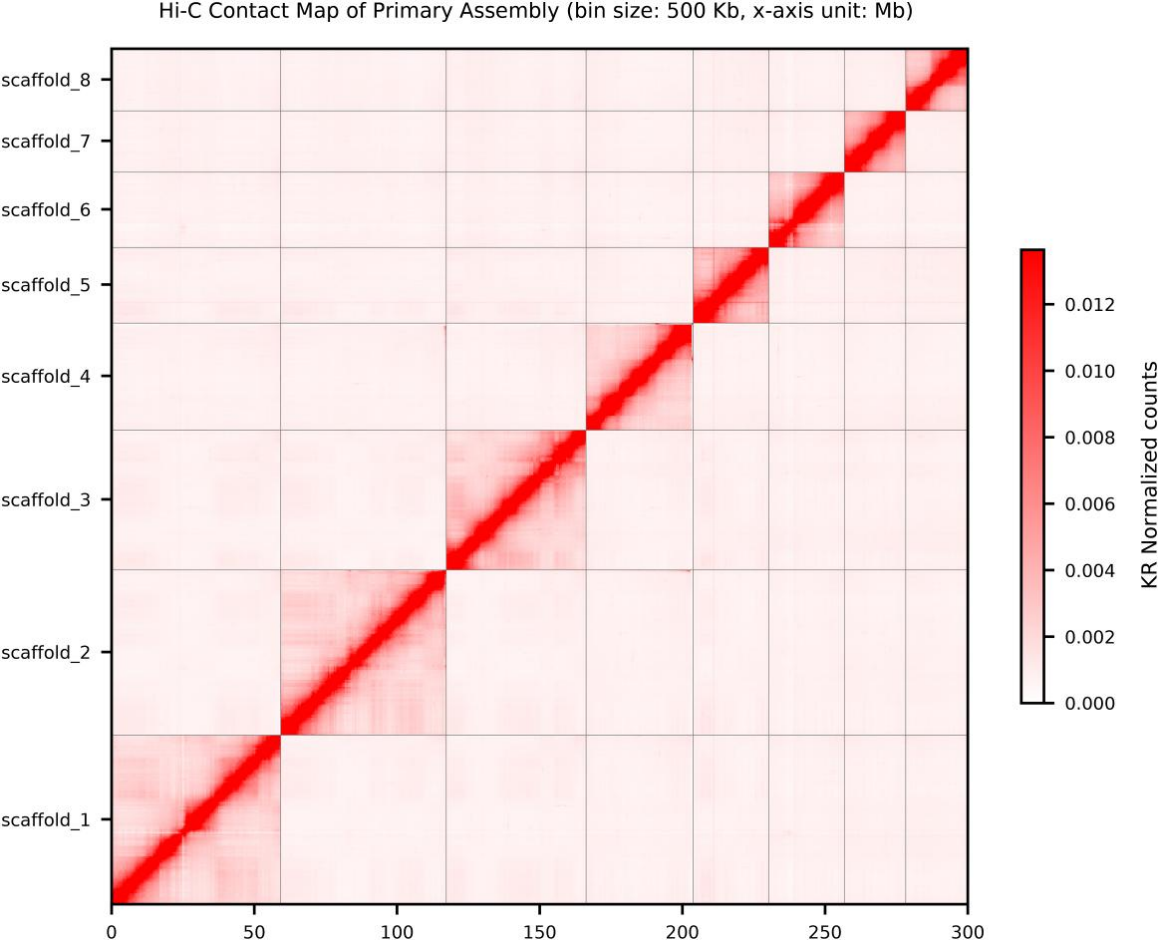
Hi-C contact map of the 8 chromosome-level scaffolds of the primary *D. antillarum* assembly.

**Fig. 2.**
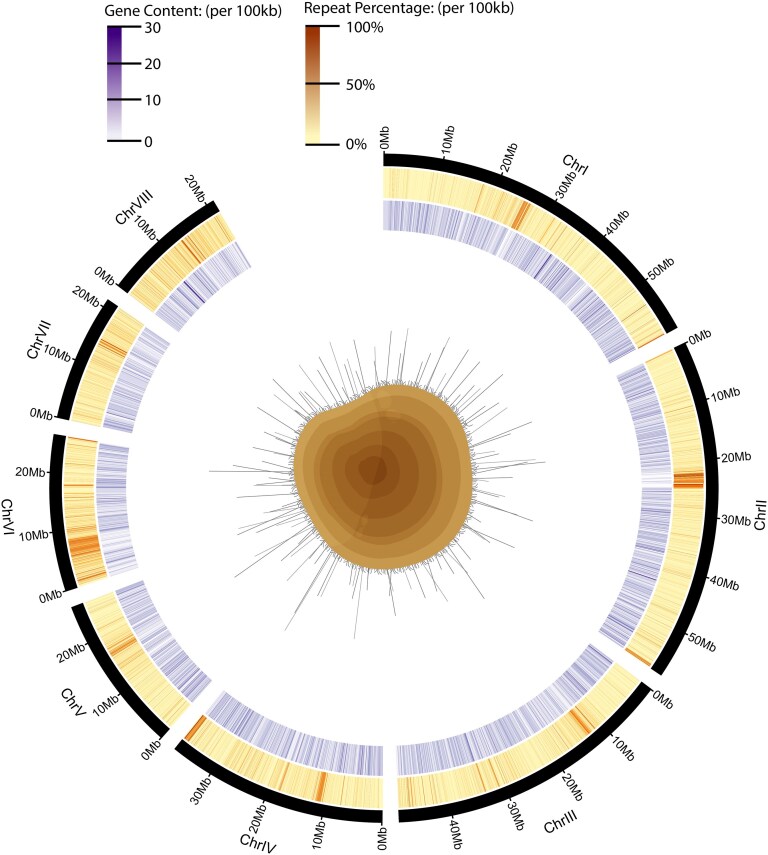
Genome assembly of *D. antillarum*. Eight chromosome-level scaffolds of the haplotype-collapsed assembly. Tick marks designate 10 Mb. From outside in: heatmap of repeat percentage (per 100 kb), heatmap of gene content (per 100 kb).

HiFi sequencing resulted in 4,581,896 HiFi reads (66,660,929,007 bp) with a mean read length of 14,549 bp and Illumina sequencing of the Hi-C library resulted in 1,521,844,217 reads (456,553 Mb). The haplotype-phased genome (Table 1) assembled into 208 scaffolds and 8 chromosome-level scaffolds (Fig. 1), with a size of 308.7 Mb, an N50 of 48 Mb, and an L50 of 3. Both haplotypes and the diploid assembly show similar levels of contiguity (Table 1; Supplementary Fig. 1). The 8 chromosome-level scaffolds contain 96.6% of all bases in the assembly. The assembly consists of 31.20% repeats and has a GC content of 36% (Fig. 2). BUSCO (genome) completeness of the primary assembly (all 208 scaffolds) based on the metazoa_odb10 dataset is 95.5% (94.4% single copy, 1.0% duplicated, 0.9% fragmented, database = metazoa_odb10) and this decreases negligibly to 95.4% (94.4% single copy, 0.9% duplicated, 1.0% fragmented) when only the 8 chromosome-level scaffolds are analyzed.

**Table 1.** Summary statistics for the *D. antillarum* genome assemblies.

Genome assembly	Primary assembly	Diploid assembly	Haplotype 1	Haplotype 2
Total length (bp)	308,701,191	608,574,745	305,238,177	303,338,868
Number of chromosome-level scaffolds	8	16	8	8
Total scaffolds	208	317	196	98
Scaffold N50 (bp)	48,854,149	48,671,606	48,440,619	48,971,102
Scaffold L50 (bp)	3	6	3	3
GC content (%)	36	35.82	35.9	35.74
BUSCO completeness (nucleotide)	95.5% [S: 94.4%, D: 1.0%], F: 0.9%	95.8% [S: 3.0%, D: 92.8%], F: 0.5%	95.6% [S: 94.8%, D: 0.8%], F: 0.8%	95.4% [S: 94.2%, D: 1.2%], F: 1.0%
Merqury completeness(%)	71.7148	99.64	71.4	71.57

The merqury (Rhie et al. 2020) completeness score (distinct k-mers filtered for low-copy k-mers in an assembly/solid k-mers in a read set) for the haplotype-phased (i.e. primary) assembly is 71.71% and it is 99.64% for the haplotype-resolved (i.e. diploid/2*N* = 16) assembly. The percentage of shared k-mers between haplotypes calculated with meryl is ∼68%, indicating high sequence divergence between haplotypes. The score of 71.71% (71.4% for haplotype 1 and 71.57% for haplotype 2) as opposed to 99.64% when both haplotypes are considered is due to haplotype 1, haplotype 2, and the haplotype phased assembly containing haplotype-specific (or haplotype phased) k-mers. The haplotype-resolved assembly score of 99.64% indicates that our assembly captures >99% of the genome's content when both haplotypes are considered.

The annotated genome contains 29,208 genes. The BUSCO (protein) completeness score (also based on the metazoa_odb10 dataset) of the translated gene models is 95.6% (87.6% single copy, 8.0% duplicated, 2.8% fragmented; Table 2).

**Table 2.** Summary statistics for the *D. antillarum* genome annotation (primary assembly).

Genome annotation:	
Genes	29,208
Repeats (%)	31.20%
Average gene length (bp)	6,199
Median gene length (bp)	3,573
Average coding sequence length (bp)	1,590
Median coding sequence length (bp)	1,131
BUSCO completeness (protein)	95.6% [S: 87.6%, D: 8.0%], F: 2.8%

S, BUSCO single-copy genes; D, BUSCO duplicated genes; F, BUSCO fragmented genes.

### Synteny

Interchromosomal rearrangements are informative for understanding genome structural evolution and evolutionary relationships (e.g. Simakov et al. 2022; Martín-Zamora et al. 2023; Schultz et al. 2023). To compare the genome organization of *D. antillarum* to that of other lophotrochozoans (and a deuterostome outgroup), we identified 1,314 single-copy genes shared among 5 species: *D. antillarum* (Brachiopoda; Dan), *P. maximus* (Mollusca; Pma), *L. longissimus* (Nemertea; Lin), *W. subatra* (Bryozoa; Wat), and *B. floridae* (Chordata; Bfl). Of these, 539 single-copy genes could be unambiguously assigned to one of the 24 bilaterian ALGs.

**Fig. 3.**
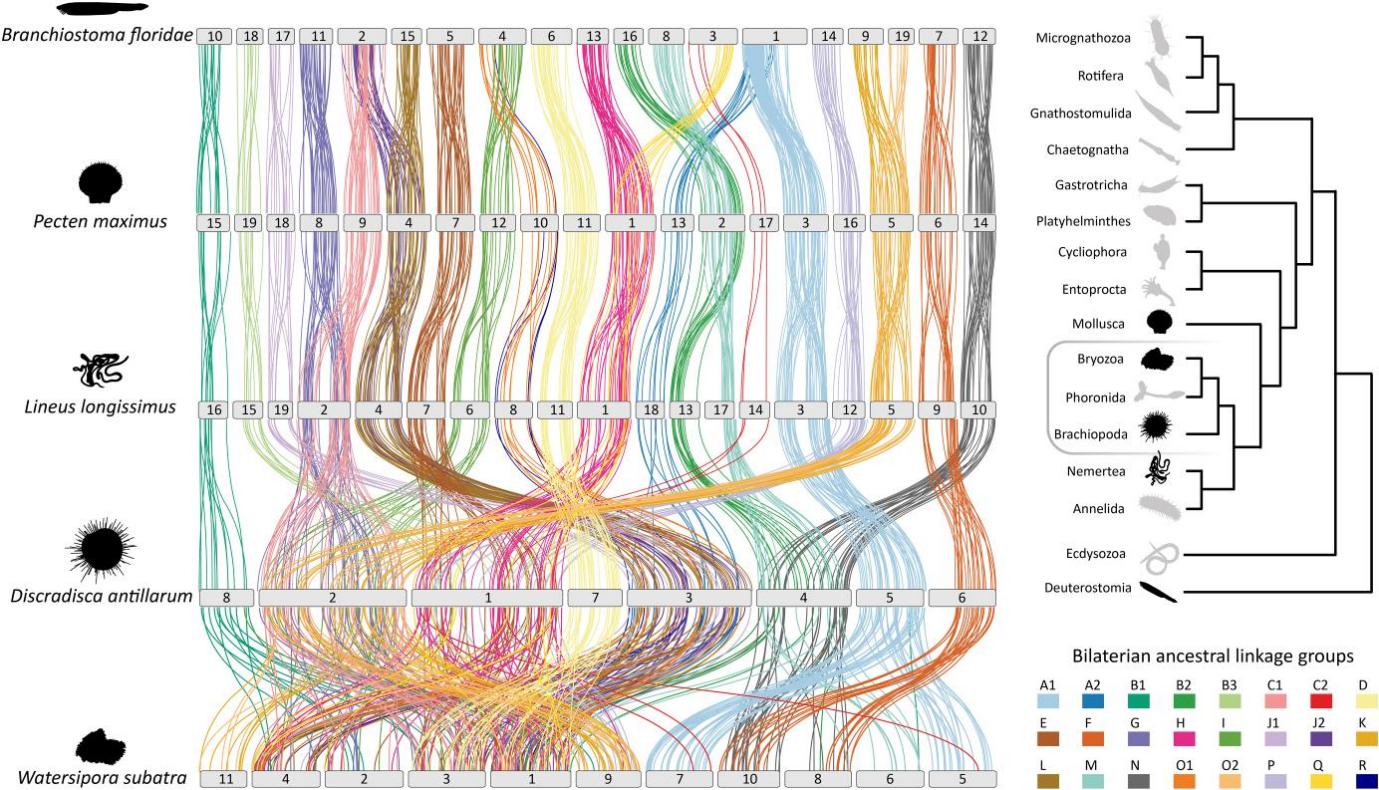
Chromosome-scale linkage of 539 orthologs assigned to bilaterian ALGs shared among *D. antillarum* (this study), *P. maximus* (Mollusca), *L. longissimus* (Nemertea), *W. subatra* (Bryozoa), and *B. floridae* (Chordata). Vertical lines connect the genomic position of orthologous genes in each genome and are color-coded based on the 24 bilaterian ALGs identified by Simakov et al. (2022).

Here, we use the algebraic notation described in Simakov et al. (2022) to describe chromosome fusion-with-mixing (⦻) and syntenic equivalence without regard to collinearity (≡). Our results are consistent with previous studies showing that the cephalochordate *B. floridae*, the scallop *P. maximus*, and the nemertean *L. longissimus* display a high degree of similarity in their organization, having largely conserved the bilaterian ALGs (Simakov et al. 2022; Lewin et al. 2025; Fig. 3). Focusing on the lophotrochozoans, we recover previously noted lineage-specific fusion events in *L. longissimus* (C1 ⦻ G) and *P. maximus* (M ⦻ B2) and the 4 proposed ancestral lophotrochozoan fusion-with-mixing events (H ⦻ Q, O2 ⦻ K, L ⦻ J2, and O1 ⦻ R; Martín-Zamora et al. 2023; Lewin et al. 2024, 2025), which are shared by all sampled lophotrochozoans including *D. antillarum*.

Within *D. antillarum*, chromosome 1 is the result of fusion-with mixing of 6 bilaterian ALGs; H ⦻ Q ⦻ C2 ⦻ P ⦻ E ⦻ B3 (Fig. 3; Supplementary Fig. 2), preserving the proposed ancestral lophotrochozoan fusion-with-mixing of ALGs H ⦻ Q. Chromosome 2 is the result of fusion-with-mixing of 5 bilaterian ALGs; C1 ⦻ G ⦻ I ⦻ O2 ⦻ K (Fig. 3; Supplementary Fig. 2), preserving the proposed ancestral lophotrochozoan fusion-with-mixing of ALGs O2 ⦻ K and, notably, sharing the fusion-with-mixing of C1 ⦻ G that is present in *L. longissimus* but not *P. maximus*. Chromosome 3 is the result of fusion-with-mixing of 6 bilaterian ALGs; L ⦻ J2 ⦻ O1 ⦻ R ⦻ J1 ⦻ A2 (Fig. 3; Supplementary Fig. 2), preserving the ancestral lophotrochozoan fusion-with-mixing of L ⦻ J2 and O1 ⦻ R. Lastly, chromosome 4 is the result of fusion-with-mixing of 3 bilaterian ALGs; M ⦻ B2 ⦻ N (Fig. 3; Supplementary Fig. 2), of which the fusion-with-mixing of M ⦻ B2 is present in *P. maximus* but not *L. longissimus*. *D. antillarum* chromosomes 5–8 are conserved with regard to the bilaterian ancestral linkage groups, with syntenic equivalency with *P. maximus*, *L. longissimus*, and *B. floridae* across several chromosomes (B1—Dan8≡Lin16≡Pma15≡Bfl10; D—Dan7≡Lin11≡Pma11≡Bfl6; A1—Dan5≡Lin3≡Pma3; F—Dan6≡Lin9≡Pma6≡Bfl7; Fig. 3; Supplementary Fig. 2).

The bryozoan *W. subatra* is the closest relative of *D. antillarum* sampled in this analysis. However, Lewin et al. (2025) showed extensive chromosomal rearrangement in Bryozoa. Like other bryozoans studied to date, *W. subatra* exhibits a high degree of rearrangement and mixing of the bilaterian ALGs relative to the other phyla sampled (Fig. 3; Lewin et al. 2025). Several fusion-with-mixing events are shared, including all proposed lophotrochozoan fusion-with-mixing events (H ⦻ Q, O2 ⦻ K, L ⦻ J2, and O1 ⦻ R; Fig. 3). Comparison of macrosynteny between *W. subatra* and *D. antillarum* suggests that multiple ancient chromosome fusions followed by gene mixing led to the near-complete loss of the bilaterian ALGs in the last common ancestor of Bryozoa after the cladogenic event that gave rise to Brachiopoda.

While several *D. antillarum* chromosomes (e.g. chromosomes 5 to 8) appear relatively conserved, further comparisons with other brachiopod genomes and additional lophophorate taxa such as Phoronida are necessary to determine the extent to which the fusion-with-mixing events observed in *D. antillarum* are lineage-specific. A preprint (Lewin et al. 2024) describing the chromosome-level genome of another brachiopod, *L. anatina*, reports that it has 10 chromosomes as opposed to the 8 found in *D. antillarum*. Lewin et al. (2024) also examined interspecies chromosomal synteny between Lingula anatina, Pecten maximus, and the cephalochordate Branchiostoma floridae. In L. anatina, chromosome 1 is the result of fusion-with-mixing of 7 ALGs (L ⦻ J2 ⦻ O1 ⦻ R ⦻ C1 ⦻ G ⦻ I ⦻ O2 ⦻ K), of which 3 of these fused pairs (O2 ⦻ K, L ⦻ J2, and O1 ⦻ R) were proposed to be ancestral for Lophotrochozoa (Lewin et al. 2025). Moreover, chromosome 2 of *L. anatina* is the result of fusion-with-mixing of 4 ALGs (H ⦻ Q ⦻ C2 ⦻ A1), of which 1 pair was proposed to be ancestral for Lophotrochozoa (Fig. 3; Supplementary Fig. 2). In contrast, the genome of *D. antillarum* has undergone multiple fusions not seen in *L. anatina* (Dan1: C2 ⦻ P ⦻ E, Dan3: J1 ⦻ A2, Dan4: M ⦻ B2 ⦻ N) (Fig. 3; Supplementary Fig. 2). Lewin et al. (2025) identified 9 fusion-with-mixing events of bilaterian ALGs shared between Bryozoa and *L. anatina*. Discradisca, however, does not share the fusion-with-mixing of A1 ⦻ C2—A1 remains conserved on *D. antillarum* chromosome 5, and C2 is located on chromosome 1 (H ⦻ Q ⦻ C2 ⦻ P ⦻ E ⦻ B3). Thus, only 8 fusion-with-mixing events are shared between Bryozoa and Brachiopoda, with the fusion-with-mixing of A1 ⦻ C2 being convergent in *Lingula* and in Bryozoa. Comparison of synteny between species in other brachiopod clades outside of the subphylum Linguliformea (Craniiformea and Rhynchonelliformea) and the phylum Phoronida with that of *L. anatina* and *D. antillarum* will be important for fully understanding the evolution of lophophorate genome organization in the phylum and lophophorate evolutionary relationships.

## Supplementary Material

jkaf233_Supplementary_Data

## Data Availability

All sequencing data related to this project have been deposited at NCBI under BioProject accession PRJNA1244359. Scripts used for genome annotation, and Hi-C scaffolding can be found at: https://github.com/ngroberts/Discradisca_antillarum/. Supplementary data including the proteomes used for annotations and Hi-C.agp and .hic and assembly files can be found on FigShare at: https://doi.org/10.6084/m9.figshare.28700648.v1. Currently, assemblies (all haplotypes and phased assembly) and annotation are available on figshare: https://doi.org/10.6084/m9.figshare.28700648.v1. Supplemental material available at G3 online.
