# Supplementary material for: Chromosome-level haplotype-resolved genome assembly of the linguliform brachiopod Discradisca antillarum (d'Orbigny, 1845)
Source: G3 (Bethesda). 2025 Oct 1;15(12):jkaf233. doi: 10.1093/g3journal/jkaf233 (PMC12693507; doi:10.1093/g3journal/jkaf233)
Supplement: jkaf233_Supplementary_Data [file jkaf233_supplementary_data.docx]

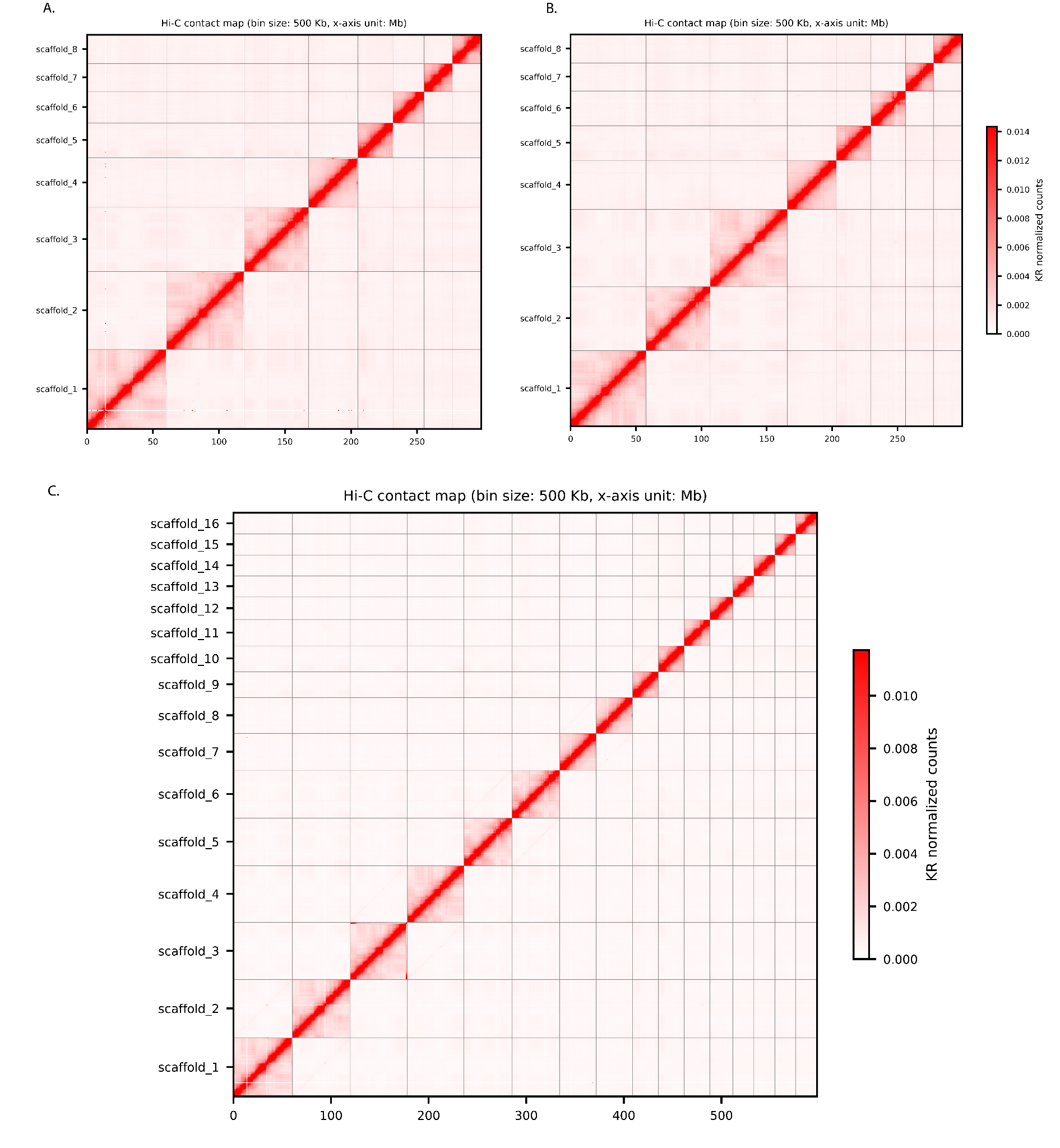


Supplementary Figure 1. Hi-C contact map of the chromosome-level scaffolds of A. Haplotype 1, B. Haplotype 2, C. Diploid assembly of *Discradisca antillarum*.


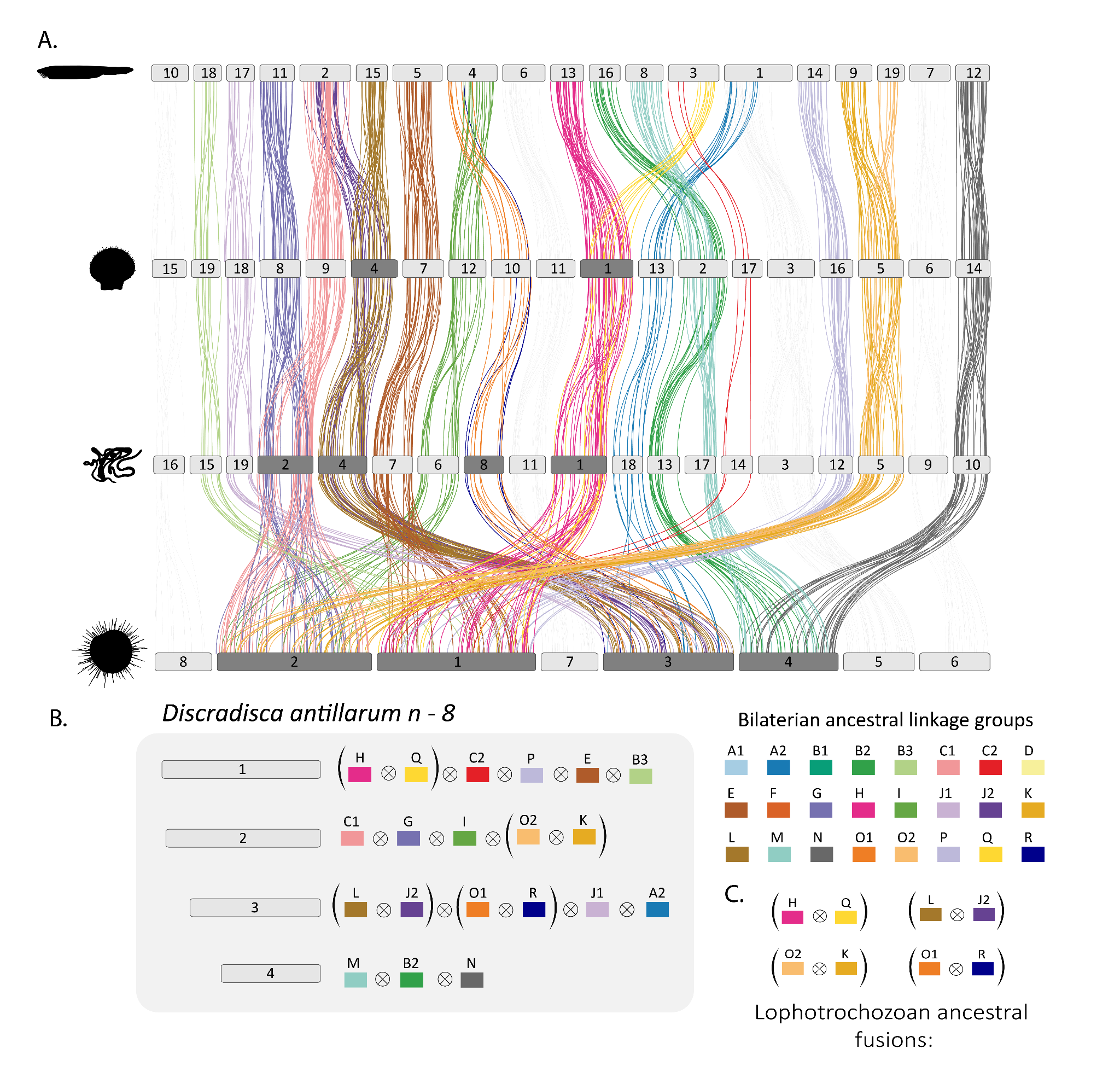


Supplementary Figure 2. A. Chromosome scale linkage of 1,133 orthologs assigned to bilaterian ALGs shared among *Branchiostoma floridae* (Chordata; GCA_000003815.2), *Pecten maximus* (Mollusca; GCF_902652985.1), *Lineus longissimus* (Nemertea; GCF_910592395.1), and *Discradisca antillarum* (this study). B. Schematic representation of fusion-with-mixing events in the chromosomes of *Discradisca antillarum*, parenthesis indicate fusions that are proposed to be ancestral in Lophotrochozoa (C).
